# Acceptability and readiness to promote human papillomavirus vaccination at ages 9-10 years: A pilot study among rural North Carolina clinics

**DOI:** 10.21203/rs.3.rs-2326137/v1

**Published:** 2023-01-30

**Authors:** Nadja Vielot, Robyn M. Lane, Kaitlyn Loefstedt, Jennifer Cunningham, Jason Everson, Eli Tiller, Sallie Patel, Jennifer Smith

**Affiliations:** The University of North Carolina at Chapel Hill School of Medicine; The University of North Carolina at Chapel Hill Gillings School of Global Public Health; Orange County Health Department; Piedmont Healthcare Inc; Piedmont Healthcare Inc; Piedmont Healthcare Inc; Piedmont Healthcare Inc; The University of North Carolina at Chapel Hill Gillings School of Global Public Health

**Keywords:** Human papillomavirus, vaccines, providers, recommendations, adolescents, rural, underserved

## Abstract

While 9-valent human papillomavirus vaccination (HPV-9) is approved by the U.S. Food and Drug Administration for use in adolescents as young as age 9, providers typically recommend it at ages 11–12 per Centers for Disease Control and Prevention recommendations. Studies suggest that recommending HPV-9 at 9 or 10 years of age could increase up-to-date vaccination by age 13, which could benefit rural populations with reduced access to primary health care and lower HPV-9 coverage than urban areas. This pilot study aimed to assess the feasibility of earlier recommendation of HPV-9 in rural clinics. We conducted in-depth interviews with providers and staff from two primary care clinics in central North Carolina, to understand attitudes toward recommending HPV-9 to 9- and 10-year-olds. All interviewees agreed that HPV-9 was important for cancer prevention and should be recommended before the onset of sexual activity, and agreed that HPV-9 could be initiated before age 11 to improve timeliness and completion of the vaccination series. However, opinions were mixed on whether it should be initiated as young as 9-years-old. Two key informants recruited from two urban clinics described their experiences recommending HPV-9 to 9- and 10-year-olds, including a modified vaccination schedule that promotes HPV-9 during routine well-child visits, prior to pubertal onset, and alongside other recommended adolescent vaccines. Earlier recommendation and administration of HPV-9 is possible with minimal changes to current clinical practices and could increase convenience and acceptability of HPV-9 in under-vaccinated settings.

## Background

Two doses of 9-valent human papillomavirus vaccine (HPV-9) administered 6–12 months apart can prevent up to 90% of cervical cancers and large proportions of vulvar, vaginal, anal, and of head and neck cancers caused by persistent high-risk HPV infection.^1,2^ However, in 2020, less than half (45.6%) of adolescents in the United States had completed the HPV-9 series on time, defined by the Centers for Disease Control and Prevention (CDC) as before their 13th birthday.^3,4^ Furthermore, HPV-9 rates are disproportionately lower among rural adolescents, who are up to 25% less likely than urban adolescents to initiate or complete the HPV-9 series.^5^ Compared to cities, rural areas tend to also have higher cancer incidence and mortality, including for cervical cancer.^6,7^ As such, improving HPV-9 rates is a priority in the rural areas.

While the Food and Drug Administration (FDA) has approved HPV-9 for adolescents starting at age 9 based on safety and efficacy data from clinical trials,^8^ recommendations about when providers should begin recommending HPV-9 differ across leading health organizations. The CDC routinely recommends initiating HPV-9 at ages 11–12 as part of an adolescent vaccination package with tetanus-containing (Tdap) and quadrivalent meningococcal (MenACWY) vaccines, and defines “up-to-date” vaccination as receipt of two doses by age 13. In contrast, the American Cancer Society and American Academy of Pediatrics have recently recommended routine initiation at age 9,^9,10^ while the CDC only allows discretional use of HPV-9 for those ages 9-10.^11^ Studies from urban centers have shown that adolescents who initiate HPV-9 at ages 9–10 are more likely to be fully vaccinated by age 13, compared to those who initiate at age 11 or older.^12,13^ A benefit of earlier HPV-9 to 9- and 10-year-olds includes allowing more time to complete the vaccination series by age 13 and before the initiation of sexual activity, which may be beneficial to adolescent who experience health care disruptions or reduced access to routine preventive care.

While early HPV-9 findings are promising, questions remain about the feasibility of translating these findings into health system change, specifically in rural communities where the need is greatest. To effectively implement early HPV-9 recommendations, clinics must be able to monitor vaccination rates of 9- and 10-year-olds, and vaccine providers must be willing to recommend and provide HPV-9 to this younger age group. In a recent national survey, only 21% of primary care professionals reported that they routinely recommend HPV-9 at ages 9–10; however, 61% reported being willing to start recommending earlier, with no differences in willingness between urban and rural providers.^14^ Thus, an earlier HPV-9 strategy could possibly be feasible in rural settings where provider willingness is high.

With a rural population of approximately 2 million people,^15^ North Carolina’s (NC) offers opportunities to test HPV-9 promotion strategies in rural populations with reduced access to cancer screening and treatment. This pilot study aimed to assess the feasibility of earlier recommendation of HPV-9 in a small sample of NC clinics that provide HPV-9 to adolescents and serve rural populations. Findings from this pilot study will inform the design of larger and more in-depth studies to assess clinic workflow around HPV-9 administration, feasibility of recommending HPV-9 uptake, and developing clinic-based interventions to recommend earlier HPV-9 initiation and potentially increase up-to-date HPV-9 coverage in rural NC.

## Methods

### Study sites and participants

We actively recruited from two NC clinics that serve rural populations as part of a system of federally qualified health centers that serve patients throughout central NC. The study was reviewed and approved by health system leadership, and clinic-based leaders identified clinicians and other clinic personnel to complete interviews. Eligible clinics recommended routine HPV-9 to adolescents at ages 11–12 years and served communities with majority-rural residents. Eligible interviewees within clinics included any provider who discussed HPV-9 with patients and caregivers, medical staff who administered HPV-9, and administrative staff who monitored HPV-9 rates, conducted patient outreach for HPV-9, or scheduled HPV-9 visits.

In addition, we identified providers at urban practices in a neighboring academic health system who indicated that they were already recommending and providing HPV-9 to 9- and 10-year-old patients during the course of a separate research study on HPV-9. We invited these providers to participate in *post hoc* in-depth key-informant interviews to describe their experiences and best practices for recommending earlier HPV-9.

### Rural clinic and patient characteristics

We asked a clinic administrator or a lead provider in each rural clinic to respond to a questionnaire describing characteristics of their clinics and patients, and clinical practices around HPV-9. Using existing EHR queries developed and validated by clinic IT staff, we estimated the number of active patients eligible for two-dose HPV-9 (i.e., patients ages 9–14 who completed a routine medical visit (i.e., well-child check) in the clinic in the last 18 months) and the proportion who had initiated or completed the HPV-9 series in each clinic.

### Development of the interview guide

The research team, which included members with expertise in public health education and communication, developed the interview guide (Appendix 1) using an iterative process. We created questions based on a literature review identifying key themes around clinical practices for adolescent and early HPV-9, in accordance with the study aim.^16-19^ The guide was reiteratively developed in collaboration with a master’s-level Qualitative Research Specialist in implementation science and reviewed by a primary care provider with over 20 years of experience in family medicine and maternal and child health. The guide probed rural clinic personnel on the perceived importance of HPV-9, the messages used to recommend HPV-9, willingness to promote early HPV-9, and the perceived facilitators and barriers to promoting early HPV-9.

Key informant interviews were conducted *post hoc*, after learning that they were already recommending HPV-9 to their younger patients. The same interview guide that was used for rural clinic personnel interviews was used for key informant interviews, though some questions were modified to ask key informants specifically about their process for recommending HPV-9 to 9- and 10-year-old patients, rather than their willingness to do so (Appendix 2). Key informants were also asked to assess feasibility of implementing earlier HPV-9 on a larger scale, including in clinics with fewer human and technological resources.

### In-depth Interviews

Thirty-minute interviews were conducted via Zoom by the study Principal Investigator (NAV) and a qualitative research assistant (RML) between April and July 2022. This study of provider practices and attitudes toward HPV-9 was determined not to constitute human subjects research by the Institutional Review Board of the University of North Carolina at Chapel Hill (UNC) (IRB #: 21–0182). As such, written informed consent was not required, and interviewees were instead emailed a description of the study design ahead of time, informing them of the nature of the interview prompts and the fact that interviews would be digitally recorded. Interviewees verbally consented to audio and video recording at the start of each interview prior to initiating the recording and received $50 Amazon gift cards for completing the interview. Zoom’s auto-transcription feature was used to generate preliminary transcripts. The research assistant then reviewed and corrected auto-transcription errors for each interview to reflect the content of the interview based on audio and video recordings.

### Analysis

We used a rapid assessment approach to identify overarching patterns and used thematic content analysis to summarize key findings. To address our research aim of assessing the feasibility of earlier recommendation of HPV-9 in rural clinics, we identified and agreed upon an initial set of topical codes and interpretive codes using preliminary memos and discussion of themes. A codebook was developed to further define each code prior to coding all the transcripts. We used the online Dedoose software version 9.0.54 (SocioCultural Research Consultants, Los Angeles, CA) to apply topical codes to excerpts from qualitative transcripts. To address inter-rater reliability, two study staff (NAV, RML) independently coded two different transcripts. Frequency and consistency of code application between the study staff was compared using the Code Application feature in Dedoose and discussed between the study staff to increase consistency between coders and increase the internal validity of the study analysis. In the first two transcripts, the two study staff had 65.5% agreement in code use. After resolving coding discrepancies, we fine-tuned the codebook to indicate more specifically when to apply and not to apply each code. While this pilot study did not intend to reach saturation of themes from a small number of interviews, we identified several themes that were reported by multiple interviewees and report these in the main results.

## Results

### Interviewee and clinic characteristics

We completed a total of 10 in-depth interviews with personnel from two rural clinics, including 2 providers, 4 nursing staff, 3 medical assistants, and 1 practice manager (Table 1). We also completed two in-depth interviews with key informants who were both medical providers in urban academic clinics; one provider was employed at a family medicine clinic, and the other was employed at a pediatrics clinic (Table 1).

Rural clinic A was located in a county where most residents lived in rural areas, in contrast to rural clinic B (Table 2). Both clinics had a family medicine focus, though rural clinic B additionally employed pediatricians and served a larger pediatric population. Both clinics had standing orders for HPV-9, though only rural clinic A performed routine patient outreach to schedule vaccination appointments, in part because of a smaller clinical and support staff. Neither clinic used a script or specific language to recommend HPV-9. Rural clinic B, having a pediatrics focus, served over twice as many patients ages 9–14 as rural clinic A. Most patients in both clinics received Medicaid or other public insurance, and both clinics had a substantial proportion of uninsured patients.

Both clinics had comparable proportions of patients with documented HPV-9 in their EMR (Rural clinic A: 47%; Rural clinic B: 48%), and similar distributions of HPV-9 initiation and completion by current patient age (Table 2). Completion of two doses tended to be higher among children who initiated vaccination at ages 9–10 compared to ages 11–12 in both clinics (Rural clinic A: 71% vs. 59%, p = 0.02; Rural clinic B: 78% vs. 65%, p = 0.5). In both clinics, 100% of patients who initiated vaccination at ages 9–10 completed the series by age 13, compared to 91% who initiated at ages 11–12.

### Thematic content analysis – Rural clinic personnel

We identified four predominant themes from the interviews: 1) clinics have created opportunities to recommend HPV-9 during well-child visits; 2) providers educate caregivers who are hesitant about HPV-9; 3) providers often consider the benefits of HPV-9 in the context of adolescent social and physical development; and 4) providers are generally willing and able to promote earlier HPV-9 in the clinic.

### Existing opportunities to recommend HPV-9

Interviewees at both clinics reported that HPV-9 is standard of care for adolescents, and predominantly occurs during routine well-child visits. In preparation for well-child visits, a medical assistant (MA) will review the patient’s EMR to identify any gaps in vaccination according to the CDC’s recommended vaccine schedule. MAs also review the North Carolina Immunization Registry (NCIR), where use of state-purchased vaccines is required to be reported, to review patient eligibility for vaccination. Alerts are triggered in both systems at age 11, when a patient is eligible to receive adolescent vaccines per CDC recommendations.

Though not routine, vaccinations are occasionally offered during sick visits to take advantage of the opportunity:

“There are times where kids come in for non-well-child checks and are due for vaccines…. sometimes people will be like well, it's not a well-child check, so we're not going to do vaccines. I'm like well, they're here.” (Rural clinic A, Clinic Staff)

In addition, both clinics offer “nursing only” visits in which patients can receive vaccines without a provider consultation.

### HPV-9 education for hesitant parents

Interviewees at both clinics reported that they rarely encountered hesitancy from caregivers to vaccinate their adolescent against HPV. However, interviewees had difficulty estimating their adolescent vaccination rates, and perceptions of the need to improve HPV-9 practices differed even within clinics.

“We have no idea. We see a lot of adolescents.” (Rural clinic B, Provider)

"I guess I have not had anyone that I remember that refused the HPV-9.” (Rural clinic A, Provider)

“We need to work on the HPV-9 rate.” (Rural clinic A, Clinic Staff)

When asked how they handled HPV-9 hesitancy or questions from caregivers, all interviewees responded that they educate caregivers on the purpose of HPV-9 and its safety profile. The most common messages include the vaccine’s role in preventing HPV-associated cancers later in life.

“Mostly it's in the 30s that we're seeing positive [HPV tests] for women, and I saw one male have a positive, so they're like your child can get this later in life. We can vaccinate now to prevent.” (Rural clinic A, Provider)

“[HPV is] a virus that causes really gross warts in your private area, but it can also lead to cancer later… we know that any person can get cancer in the future.” (Rural clinic B, Clinic Staff)

At times, an MA will brief the caregiver and patient on vaccines that are due, and they will report to the provider if any hesitancy was expressed. Providers reported that they were often able to persuade a hesitant or questioning caregiver to accept HPV-9 following a brief discussion of the benefits of the vaccine. Other times, caregivers have misconceptions about the HPV-9, which the provider must then correct.

“if the provider [is] still kind of reviewing the next patient’s chart, I’ll kind of just get them real quick, I’m like hey they have questions, concerns. Because some parents will say yes to the provider.” (Rural clinic B, Clinic Staff)

“Sometimes they’re like, ‘well he’s a male, he won’t get it.’ I said, ‘you’re wrong because it can affect males as well as females.’” (Rural clinic A, Clinic Staff)

### HPV-9 relates to social and physical development

The child’s age and developmental stage was a common consideration among interviewees when discussing HPV-9 with caregivers. Several reported that caregivers expressed that HPV-9 was not appropriate for their adolescent, either because they are not yet sexually active and not at risk for HPV, or because they perceived HPV-9 as an enticement to initiate sexual activity. Several interviewees reported that they had children or grandchildren of their own and would want them to be protected against HPV-associated cancers.

“I’ve certainly heard of people being hesitant to receive the vaccine because of concern regarding sexual promiscuity at a young age.” (Rural clinic A, Clinic Staff)

“I’m a mom. I have three kids, one of them is a male and two females. I really want them to be as protected as they can be.” (Rural clinic A, Clinic Staff)

When asked for their opinions on the ideal age to initiate HPV-9, all interviewees reported that 11–12 years (n = 2) or younger (n = 7) was ideal. The common justification was that HPV-9 should be given prior to the onset of sexual behavior to be most effective. Some interviewees reported that the youngest eligible age (e.g., 9 years) was ideal, as they had heard of or encountered adolescents who became sexually active before age 11.

### Providers are willing to implementing earlier HPV-9

When asked if they would be willing to recommend HPV-9 at ages 9–10 years, some interviewees themselves expressed hesitancy. Some believed that age 9 was too young, and that age 10 or older was sufficiently early to initiate HPV-9.

“… It does feel a little bit funny and maybe that's because I have children in the single digits. I don't want my child to be a teenager at 9…” (Rural clinic A, Provider)

"Ten and 11 go to where you're going to middle school now and they are hearing more and more about it. 9, I think it is too young. It’s only one year difference, but I don't know, it sounds young.” (Rural clinic B, Clinic Staff)

Some interviewees initially questioned the benefits of earlier vaccination. When the interviewers provided some probes), interviewees tended to support earlier vaccination. Interviewees also generally agreed that earlier vaccination could be implemented in their practices with additional training and some minimal changes to current practices. However, some expressed reservations, suggesting that staff are not always amenable to change and that it could be difficult to convince all personnel to embrace earlier HPV-9.

"I will say more training and acknowledgement of this vaccine would be a really nice factor. Like reeducate staff on how to approach when it comes to offering a vaccine and using the word ‘highly recommended.’” (Rural clinic A, Clinic Staff)

“I’ll be honest, at our site… change is hard here.” (Rural clinic A, Clinic Staff)

### Thematic content analysis – Key informants

Two key informants, one physician in a pediatrics practice (urban clinic A) and one in a family practice (urban clinic B) (Table 1), reported beginning discussions about HPV-9 with their 9- and 10-year-old patients. The pediatrician reported that the shift to vaccinating younger patients was a response to the clinic’s quality improvement (QI) metrics, and that initiating discussions about HPV-9 earlier at ages 9 and 10 increased the number of patients who received on-time vaccination according to clinic records.

Other providers in the practice also adopted this strategy, in part to receive monetary incentives for having high metric scores. However, adoption was not universal, and a provider hesitated to support practice-wide mandates for early vaccination:

“And so, I’ve been reluctant to have a practice-wide policy on timing [sic: age] about administration. I think it's fine to have a practice-wide policy on promotion.” (Urban clinic A, Provider)

This provider implemented an alternative vaccination schedule that retained the CDC-recommendation to co-administration of HPV with Tdap and Men4 vaccines, but allowed for earlier completion of the series, more time to complete the series on time, and fewer vaccinations per visit. When asked about challenges to implementing earlier HPV-9 across the practice, this provider mentioned very few barriers apart from having support from all providers. It was expressed that monitoring vaccination at ages 9–10 was easy as long as vaccinations were aligned with annual well-child visits:

“That’s not hard… We try to do well visits on or after the birthday. With 6 to 12 months between doses one and two, if you just give it at your 10-year-old well-visit and then your 11-year-old well-visit, you're fine, you don't really need to track [the timing between doses].” (Urban clinic A, Provider)

A family medicine provider reported recommending earlier vaccination in response to the possibility of early sexual initiation. They also reported using every clinical opportunity to encourage vaccination, usually in advance of the CDC-recommended timeline:

“The more times you mentioned something, the more likely someone is to be comfortable with it and just get it done… I talk about vaccines at almost every visit for everybody….” (Urban clinic A, Provider)

In contrast to the pediatric practice, one interviewee noted a limitation to implementing an earlier vaccination approach in a family medicine practice given the small number of adolescent patients and less urgency to adapt their existing systems.

“It's difficult because if you don't have as many kids, then you don't really have the structure to make it really easy for parents.” (Urban clinic B, Provider)

This provider suggested earlier vaccination could reasonably be incorporated into current practice, and that modifications to existing EMR-based alert systems that provide notices when a patient is overdue for recommended vaccines would facilitate earlier vaccination.

“So, if it doesn't add to the number of visits, then it seems to make sense to just do [HPV-9] at 9 and 10 and you can kind of get that over with, and then at 12, then they get their next you know set of vaccines… If they need time to think about it, then they have time to think about it. So, I feel like it makes sense to talk about it earlier rather than later.” (Urban clinic B, Provider)

“I do think that, if the [EMR alerts] fired at age 9 and said… your patient is now due for HPV, it probably would make a difference… At least we give the vaccination information sheet at that visit and then they have it ahead of time.” (Urban clinic B, Provider)

## Discussion

In interviews with providers and staff from clinics serving rural adolescent populations, we found a high level of support for earlier HPV-9 at ages 9 and 10 years of age. Interviewees suggested that their clinic procedures could reasonably be adapted to start recommending vaccine earlier than the CDC-recommended 11–12 years, with additional staff education needed. While EMR data from participating clinics show that fewer than half of patients had completed the HPV series by age 13, there is evidence that patients who initiated earlier were more likely to have completed vaccination, and to have completed on time. According to interviews with key informants from urban academic settings, some providers are already successfully providing HPV-9 earlier, and lessons learned can possibly be adapted to other primary care settings to improve the coverage of on-time HPV-9.

One important consideration of earlier HPV-9 recommendations is whether clinic personnel are aware that it can be administered to 9- and 10-year-olds. Prior studies on clinic personnel experiences with earlier HPV-9 did not measure their initial awareness of this option, which might have implications for its perceived acceptability.^12,13,20,21^ In our study, two interviewees were not aware that 11 years was not the minimum age for HPV-9, suggesting that they had not previously considered vaccinating younger patients and that they had no preconceived notions about the practice. Furthermore, even among interviewees who were aware of the FDA approval for 9- and 10-year-olds, several could not initially describe any benefits to earlier HPV-9. Continuing education for providers and clinic staff should emphasize not only that HPV-9 is indicated for younger patients, but also that emerging data suggest that it can lead to better on-time coverage and optimal prevention.

The two key informants from two academic clinics were in different stages of implementation of earlier HPV-9 vaccination: one had developed a protocol for early vaccination, including an alternative vaccine schedule, whereas the other simply introduced HPV-9 earlier. These differing approaches could reflect differences in the perceived importance of tracking adolescent vaccination rates between pediatrics and family practices. Tracking patient vaccination rates using EMR or immunization registries is an evidence-based practice to motivate providers to vaccinate their patients in a timely manner,^22-26^ and only the pediatrician key informant emphasized QI metrics as a motivator to vaccinate earlier. In contrast, family practitioners tend to see fewer adolescent patients than pediatricians and are possibly less likely to report or fulfill QI measures related to adolescent vaccines, including HPV-9.^27-29^ While this pilot study cannot draw firm conclusions on the differences in motivations between pediatrics and family practices, more research on this topic is warranted.

Some practice-based challenges make it difficult to start recommending and administering HPV-9 at ages 9–10. Interviewees reported relying on notifications from EMR or NCIR to determine when a patient was due for vaccination. While NCIR indicates an “earliest date” for vaccination at age 9, based on the patient’s recorded age, the “recommended date” is age 11 in accordance with CDC recommendations.^30^ Changes to these recommended ages identified by these alert systems might impel staff and providers to recommend vaccination to 9- and 10-year-olds without making any additional efforts. This successful change has been documented in a prior study of QI measures in a primary care network based in Columbus, Ohio, and more information is needed to understand how software programming decisions and modifications can be made in different clinic systems.^12^

This pilot study included a small number of clinics, all of which were affiliated with or proximal to a large, well-resourced academic center, and do not represent the experiences of clinics in more remote areas and with fewer pediatric providers. In addition, all participating clinics had sophisticated EMR that could be programmed to indicate when vaccines were due. One limitation of this data is that clinic EMR do not capture vaccinations that happened outside of the clinic system, and vaccination coverage based on EMR is likely underreported. Clinics with more rudimentary systems or paper records would have to identify other strategies for identifying vaccine-eligible 9- and 10-year-olds and monitoring vaccine receipt. However, the alternative vaccination timeline proposed by one of the key informants (i.e., providing HPV and Tdap vaccination at age 10, and HPV and Men4 vaccination at age 11 during annual well-child visits), could be implemented in any primary care clinic, with no need for additional tracking or alerts.

HPV-9 of 9- and 10-year-olds carries several advantages and could be facilitated with provider education and support. However, while modifications to vaccination schedules can be implemented without major disruptions to current practice, it can be difficult to build support for such changes in settings with high patient volumes and limited time. This exploratory qualitative study provides insight regarding the current status of how HPV-9 vaccine is perceived by providers in real world settings and allows the research team to find effective methods to promote earlier HPV-9 as an effective, convenient, and acceptable practice for patients in rural settings.

## Conclusions

An earlier vaccination practice could be piloted in a small number of clinics that are willing and able, and lessons learned could be used to make practice-specific improvements to earlier vaccination practices. Findings from these studies could support effectiveness trials of a novel early HPV-9 intervention, and recommendations for implementing early vaccination in a variety of settings.

## Figures and Tables

**Table 2.** Characteristics of two rural North Carolina clinics that provide HPV-9 to adolescents

	Rural clinic A	Rural clinic B
County	Chatham	Alamance
Rural residents in the county (N, [%])	41,864 (65.9)^31^	47,561 (28.4)^32^
Primary provider specialty	Family Medicine	Family Medicine, Pediatrics
Number of health care providers (number full-time)	18 (6)	8 (5)
Number of medical staff (nurses, medical assistants)	12	7
Clinic performs routine patient outreach for HPV-9	Yes	No
Clinic has standing orders for HPV-9	Yes	Yes
Clinic uses script or guiding language for HPV recommendation	No	No
Active patients ages 9-14 years^1^	414	966
Active patients preferring care in a language other than English (N, [%])	277 (67)	622 (36)
Payer mix of active patients (N, [%])		
-Medicaid	219 (53)	690 (71)
-Health Choice/CHIP	33 (8)	12 (4)
-Private	25 (6)	60 (6)
-Uninsured	137 (33)	176 (18)
Active patients with ≥1 HPV dose documented in electronic medical record (N, [%])		
-Age 9	0 (0)	1 (1)
-Age 10	0 (0)	7 (4)
-Age 11	11 (21)	48 (35)
-Age 12	54 (67)	122 (79)
-Age 13	63 (79)	170 (85)
-Age 14	68 (80)	120 (88)
-Total	196 (47)	468 (48)
Active patients with 2 HPV doses documented in electronic medical record (N, [%])		
-Age 9	0 (0)	0 (0)
-Age 10	0 (0)	4 (3)
-Age 11	1 (2)	12 (9)
-Age 12	10 (12)	49 (32)
-Age 13	27 (34)	97 (48)
-Age 14	43 (51)	87 (64)
-Total	81 (20)	249 (26)

1 Active patients include those with a well-child check in the last 18 months, as of July 22, 2022. Patient age reflects their age at the time of the query.

**Table 3:** Major themes and illustrative quotations identified from in-depth interviews (n = 10)

Area of inquiry	Theme	Quotations
Current clinical approaches to adolescent HPV-9	Existing opportunities to recommend HPV-9	“Usually, we go by whatever NCIR is recommending, like the state of North Carolina registrations recommendation. And every time I pull a record for one of our patients, I just look and see if they have already gotten [vaccinations] or are due soon.” (Rural clinic A)
		“At any pediatric visit, we’ll print NCIRs, and so we try to pay attention at any visit to be able to offer [the HPV-9].” (Rural clinic B)
	Addressing caregiver concerns and continuing education	“And even if they still have questions, we get the provider… even if they’re with another patient, they go back into answer the parent’s questions.” (Rural clinic B)
		“I feel like some parents, they feel like we're administering the disease into their child.” (Rural clinic B)
Receptiveness to promoting HPV-9 among 9- and 10-year-olds	Considering HPV-9 and child social and physical development	“I just think the parents aren't ready to think that their kids are growing up.” (Rural clinic B)
		“Looking at my grandchildren, after five, they’re not really getting any shots. So, if they're still coming for the well-child check, go ahead and plant that seed [regarding HPV-9].” (Rural clinic A)
	Assessing provider feelings of early vaccination	“The vaccine works better the younger we give it.” (Rural clinic B)
		“I think earlier is better… you just can't predict when like kid will have that first experience with someone. Some 12-year-olds are really far away from that, and other 12-year-olds are not, and so I think that if you wait, it's just risky.” (Urban clinic B)
		“I think if you have a year of hesitancy, having that year earlier is going to make it more likely to stay on time.” (Urban clinic A)
	Willingness and ability to promote earlier HPV-9 in the clinic	“… It sort of fit neatly into a new schedule to do the Tdap and the HPV at 10, and then the HPV and the [Men4] at 11.” (Urban clinic A)
		“I can see where that would be really excited to be part of [an earlier vaccination pilot trial]…then we'd have those results, and we can say this is better.” (Rural clinic B)

## Key Messages Regarding Feasibility

- It is unknown if clinics that provide human papillomavirus (HPV) vaccination to adolescents in rural areas of the United States are willing or able to recommend HPV vaccination starting at age 9, per US Food and Drug Administration licensure.
- In 10 interviews with clinic personnel, we determined that rural clinic personnel were largely willing to recommend HPV vaccination to 9- and 10-year-old patients, but some expressed hesitancy about the need to vaccinate at age 9 and the feasibility of adapting current clinic practices.
- Two key informants with experience recommending HPV vaccination at ages 9-10 years provided insight on best practices to integrate this change into clinic practice, which can be evaluated in future randomized trials.

## Abbreviations

CDC: US Centers for Disease Control and Prevention
EMR: Electronic medical record
FDA: US Food and Drug Administration
HPV: Human papillomavirus
HPV-9: Nine-valent human papillomavirus vaccine
MA: Medical assistant
MenACWY: Quadrivalent meningococcal conjugate vaccine
NC: North Carolina
NCIR: North Carolina Immunization Registry
QI: Quality improvement
Tdap: Tetanus-diphtheria-acellular pertussis vaccine
